# Cardiovascular risks and elevation of serum DHT vary by route of testosterone administration: a systematic review and meta-analysis

**DOI:** 10.1186/s12916-014-0211-5

**Published:** 2014-11-27

**Authors:** Stephen E Borst, Jonathan J Shuster, Baiming Zou, Fan Ye, Huanguang Jia, Anita Wokhlu, Joshua F Yarrow

**Affiliations:** Geriatric Research, Education and Clinical Center, Malcom Randall VA Medical Center, 1601 SW Archer RD, Gainesville, FL 32605-1197 USA; Research Service, Malcom Randall VA Medical Center, Gainesville, FL USA; Medicine Service, Malcom Randall VA Medical Center, Gainesville, FL USA; Department of Applied Physiology & Kinesiology, University of Florida, Gainesville, USA; Department of Biostatistics, University of Florida, Gainesville, USA; Department of Health Outcomes and Policy, University of Florida, Gainesville, USA; Department of Medicine-Cardiovascular Division, University of Florida, Gainesville, USA

**Keywords:** Testosterone, DHT, Cardiovascular disease trials, Random effects, Meta-analysis

## Abstract

**Background:**

Potential cardiovascular (CV) risks of testosterone replacement therapy (TRT) are currently a topic of intense interest. However, no studies have addressed CV risk as a function of the route of administration of TRT.

**Methods:**

Two meta-analyses were conducted, one of CV adverse events (AEs) in 35 randomized controlled trials (RCTs) of TRT lasting 12 weeks or more, and one of 32 studies reporting the effect of TRT on serum testosterone and dihydrotestosterone (DHT).

**Results:**

*CV risks of TRT*: Of 2,313 studies identified, 35 were eligible and included 3,703 mostly older men who experienced 218 CV-related AEs. No significant risk for CV AEs was present when all TRT administration routes were grouped (relative risk (RR) = 1.28, 95% confidence interval (CI): 0.76 to 2.13, *P* = 0.34). When analyzed separately, oral TRT produced significant CV risk (RR = 2.20, 95% CI: 1.45 to 3.55, *P* = 0.015), while neither intramuscular (RR = 0.66, 95% CI: 0.28 to 1.56, *P* = 0.32) nor transdermal (gel or patch) TRT (RR = 1.27, 95% CI: 0.62 to 2.62, *P* = 0.48) significantly altered CV risk. *Serum testosterone/DHT following TRT*: Of 419 studies identified, 32 were eligible which included 1,152 men receiving TRT. No significant difference in the elevation of serum testosterone was present between intramuscular or transdermal TRT. However, transdermal TRT elevated serum DHT (5.46-fold, 95% CI: 4.51 to 6.60) to a greater magnitude than intramuscular TRT (2.20-fold, 95% CI: 1.74 to 2.77).

**Conclusions:**

Oral TRT produces significant CV risk. While no significant effects on CV risk were observed with either injected or transdermal TRT, the point estimates suggest that further research is needed to establish whether administration by these routes is protective or detrimental, respectively. Differences in the degree to which serum DHT is elevated may underlie the varying CV risk by TRT administration route, as elevated serum dihydrotestosterone has been shown to be associated with CV risk in observational studies.

**Electronic supplementary material:**

The online version of this article (doi:10.1186/s12916-014-0211-5) contains supplementary material, which is available to authorized users.

## Background

Testosterone replacement therapy (TRT) is being utilized at a rapidly increasing rate, with 1.6 billion dollars in sales in the US in 2011 [1]. Proven benefits for older men with low testosterone (T) levels include increases in muscle strength, exercise capacity, bone mineral density (BMD), libido and insulin sensitivity [2,3]. Meta-analysis through 2010 [4-6] confirmed three adverse events resulting from TRT: 1) polycythemia, 2) an increased number of prostate-related events, and 3) a small reduction in high density lipoprotein (HDL) cholesterol. Prostate events consist of the combined incidence of elevated prostate-specific antigen (PSA), prostate biopsy necessitated by results of digital rectal exam, increased urinary symptoms and prostate cancer [4]. A meta-analysis by Calof *et al*. shows no evidence that TRT increases prostate cancer (odds ratio =1.09 with no trend toward significance), when considered as an independent outcome [4]. However, the cardiovascular (CV) risk of TRT is controversial [5,6].

Several recent reports have raised the concern that TRT may produce CV risks. In their randomized controlled trial (RCT) of transdermal T gel administration, Basaria *et al*. reported a very high incidence of CV adverse events (AEs) in treated subjects (21%) compared to placebo (5%) resulting in cessation of the trial [7]. More recently, Vigen *et al*., in a retrospective study of 8,709 hypogonadal men with a history of recent coronary angiography, reported a higher risk of the combined endpoints of myocardial infarction (MI), stroke and all-cause mortality in those who received any form of TRT (25.7%) compared to those who did not (19.9%) [8]. Another observational study by Finkle *et al*. evaluated 55,000 patients and reported a more than two-fold greater risk of MI in men who had received a TRT prescription [9]. Similarly, a meta-analysis by Xu *et al*. of CV AEs in 27 RCTs administering TRT (reported through 2012) found that TRT produced a significantly greater number of CV AEs in TRT-treated participants compared to placebo (odds ratio (OR) 1.54, 95% CI 1.09 to 2.18) and also made the disturbing observation that these AEs were under-reported in industry-sponsored studies [10]. However, the statistical methods employed in the latter study were not appropriate for low event-rate meta-analysis [11]. In contrast, Corona *et al*. [12] published a meta-analysis of 75 studies of TRT using less stringent inclusion criteria and found no evidence of CV risk (OR = 1.07 for all CV AEs; OR = 1.01 for serious CV AEs). In response to these reports, in 2014, the US Food and Drug Administration [13], the US Veteran’s Administration [14] and the Endocrine Society [15] have all issued advisories regarding CV AEs resulting from TRT.

In contrast with the above reports, some of which indicate that TRT may be associated with [8,9] or may cause [7,10] increased CV events, there is an extensive literature supporting the CV benefits of adequate levels of endogenous T and TRT. In older men, low T is associated with increased CV risk and increased all-cause mortality [16]. Several studies have shown that TRT is beneficial in populations of older men with CV disease. English *et al*. have shown that TRT improves exercise capacity in men with angina [17]. In addition, Toma and colleagues [18] have published a meta-analysis demonstrating improved New York Heart Association (NYHA) class, six minute walk time and peak oxygen consumption after TRT in men with systolic heart failure [18]. Furthermore, in a large retrospective cohort study of more than 6,000 intramuscular TRT users and matched controls, Baillargeon *et al*. reported no increase in CV events in myocardial infarction hospitalization rates in all TRT-treated subjects and reduced rates in those who were in the quartile with the highest risk factors for CV disease [19].

One potential explanation for these apparently conflicting observations is that the CV risk/benefit ratio may vary by the route of TRT administration. Testosterone can be administered by intramuscular injection of long-acting T esters, transdermally by patch or gel and orally as testosterone undecanoate (TU). Different routes of administration are typically associated with different doses, different time courses of serum androgen elevation and different relative levels of dihydrotestosterone (DHT) relative to testosterone. Transdermally and orally administered T are exposed to a high degree of 5-alpha reductase activity present in the skin [20] and liver [21], respectively, possibly increasing serum DHT relative to testosterone, which may affect CV risk. Shores *et al*. recently reported that serum DHT is independently and positively associated with incident CV disease [22], incident stroke [23], and all-cause mortality [22]. In contract, in a cohort of 1,032 elderly men followed for a median of nine to ten years, neither circulating T nor free T were associated with the latter adverse outcomes.

The main purpose of this meta-analysis was to assess whether the incidence of CV events is affected by the mode of TRT administration. Our secondary purpose was to determine if there is a differential elevation of T versus DHT based on route of TRT administration. We postulate that the latter may be a potential mechanism for differential CV effects.

## Methods

### Data sources and searches

This meta-analysis follows the Preferred Reporting Items for Systematic Reviews and Meta-Analyses (PRISMA) checklist [see Additional file 1]. Two expert authors searched for and selected the studies, agreed upon the eligibility of each study and extracted information from the selected trials (SB, FY). We systematically searched PubMed until 31 May 2014 using two search strategies: 1) (“testosterone” or “androgen”) and (random*) and “trial” and 2) “testosterone” and “clinical trials”. Studies of men, published in English were selected and the search was supplemented by a search of the World Health Organization trial registry and by a manual search of bibliographies of identified studies (SB and FY). To identify studies reporting the elevation of serum T and DHT following TRT, we performed a supplemental search using the terms: “testosterone” and “DHT” and (“injection” or “gel” or “patch” or “oral”) that included all TRT clinical trials in men because few studies report DHT concentrations before and after TRT.

### Study selection

#### CV risks following TRT

We included only placebo-controlled RCTs of TRT that reported CV related events for both the TRT and placebo arms. We excluded trials where testosterone secretion was experimentally suppressed prior to initiation of TRT because these studies do not have a true placebo group. In order to assess the long-term, rather than the acute, effects of TRT, we included only trials lasting 12 weeks or more. Initially, we intended to exclude RCTs that only reported AEs necessitating study withdrawal, however this turned out to be a fine distinction and so we included any RCT that reported CV events by treatment arm. In order to ensure that we did not include more than one study using the same data set, we checked for duplication based on authorship, study description, number of participants, and participant characteristics. Where duplication occurred, we used the report containing the most comprehensive description of AEs.

#### Elevation of serum T and DHT following TRT

Few placebo-controlled RCTs report both serum T and DHT before and after treatment. For this reason, we broadened our search to include all TRT trials that reported both serum T and DHT, before and after treatment, regardless of study duration or whether the study was blinded. We have recently shown that commercially available methods for measuring DHT by immunoassay are invalid [24]. For this reason, we also excluded one study where DHT was measured by immunoassay [25]. We also removed any duplication of studies as described above.

### Outcome

The primary outcome was composite CV events because we anticipated too few events to allow for analysis by individual event type. CV events were defined as anything reported as such in the original study. In cases where authors sent us a table of all AEs, we (SEB and AW - Cardiologist) defined CV AEs using International Statistical Classification of Disease (ICD) 10 codes [see Additional file 2]. CV events in individual studies are listed in Additional file 3. The secondary outcome was elevation of serum T and DHT following TRT administration by different routes (intramuscular, transdermal (patch or gel) or oral).

### Data extraction and quality assessment

Data for CV AEs and elevation of serum T and DHT were extracted by trial arm by SB and FY. Event classification was checked by a cardiologist (AW). Reviewers (SB and FY) used an established tool to evaluate the quality of each trial [26] [see Additional file 4 and Additional file 5]. If the trial did not report CV AEs or did not do so by treatment arm, we contacted the authors twice by email to ask for additional information [see Additional file 6]. Studies were excluded if CV event incidence could not be determined with the above method.

### Statistical methods

#### CV events analysis

It is important to note that this collection of studies involves low event-rate randomized binomial trials. Since the trials involve diverse interventions, random effects are mandatory, whether or not a Cochran Q test fails to reject homogeneity (Borenstein *et al*. [27], section titled ‘Model should not be based on the test for heterogeneity’). In addition, commonly used methods based on inverses of variance estimators such as the DerSimonian-Laird method are not valid in this arena. The Cochrane Handbook [28], section 16.9, states ‘Methods that should be avoided with rare events are the inverse-variance methods (including the DerSimonian and Laird (DL) [29] random-effects method). Xu *et al*. [10] used a fixed effects meta-regression with inverse variance weights in their previous meta-analysis, and as such, this was not an acceptable approach when event rates are low. Furthermore, compared with a random effects model, a fixed effects model makes the strong assumption that the true effect size for all studies is identical and the inference from a fixed effects model is conditional and limited to the studies included in the meta-analysis [30]. The methodology we employ is the sample size weighted random effects method of Shuster *et al*. [11], specifically designed for low event-rate meta-analysis, and which has been vetted on nearly 40,000 low event scenarios. Given the issues with low event-rate meta-analysis, ignored in Xu *et al*., it is critically important to reanalyze those data in this paper. Note that we employ RR, the estimate of the ratio of failure rates, rather than the OR, the ratio of the odds of failure. RR and OR are very similar when event rates are low, but RRs are far easier to understand.

#### Analysis of serum T and DHT levels

For laboratory levels, we used a minor modification of the patient weighted random effects method of Shuster [31], using the more conservative t-distribution (degrees of freedom = number of studies - 1) instead of the normal approximation. In our experience this is a better large sample approximation. Means are sample-weighted. The individual study fold changes were analyzed (not the ratio of the summary post-test estimate to the summary pre-test estimate).

## Results

### Study selection and characteristics for analysis of CV risks

**Figure 1:**
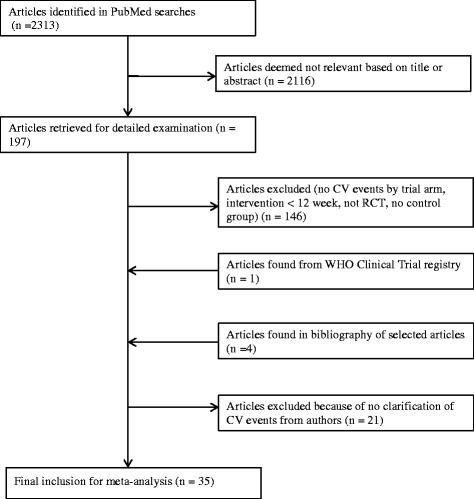
**Selection process for placebo-controlled randomized clinical trials (RCTs) of testosterone replacement therapy (TRT) on CV events.**

The initial search yielded 2,313 publications, of which 197 were subjected to further scrutiny. As shown in Figure 1, we subsequently identified 35 unique publications of placebo-controlled RCTs of TRT in men that reported CV events and met our search criteria. The study design and patient characteristics across these trials are summarized in Table 1. The 35 studies of TRT include 3,703 men, typically older than 45 years, with low T and/or chronic diseases. Of the 35 trials, 16 studies administered TRT intramuscularly, 15 transdermally (10 gel and 5 patch), and 4 orally. The mean duration of treatment was 11.9 months.

**Table 1:** **Characteristics of placebo-controlled randomized clinical trials of testosterone replacement therapy (TRT) reporting CV events**

**Author/Year**	**Mode**	**Dose**	**Study duration**	**Age**	**Subjects in TRT group**	**Subjects in placebo group**	**Serum T at entry**	**Health status**
Amory 2004 [32]	i.m.	100 mg TE/week	36 months	71 ± 4 (SD)	24	24	302 ± 48 (SD) ng/dL	hypogonadal
Aversa 2010 [33]	i.m.	1,000 mg TU/12 week	24 months	58 ± 10 (SD)	40	10	259 ± 48 (SD) ng/dL	hypogonadal
Borst 2014 [34]	i.m.	125 mg TE/week	12 months	69.2 ± 8.0 (SD)	31	29	264 ± 92 (SD) ng/dL	hypogonadal
Caminiti 2009 [35]	i.m.	1,000 mg TU/8 week	4.5 months	66 to 76	35	35	230 ± 180 (SD) ng/dL	hypogonadal, heart failure
Ferrando 2002 [36]	i.m.	100 mg TE/week	6 months	67 ± 3 (SD)	7	5	<480 ng/dL	eugonadal
Hackett 2014 [37]	i.m.	1000 mg TU/6 to 12 weeks	7.5 months	18 to 80	97	102	301 ± 11 (SD) ng/dL	hypogonadal, type 2 diabetes
Hall 1996 [38]	i.m.	250 mg TE/4 weeks	9 months	60.8 ± 9.7 (SD)	17	18	458 ± 187 (SD) ng/dL	eugonadal, rheumatoid arthritis
Ho 2011 [39]	i.m.	1,000 mg TU/10 to 14 weeks	24 months	≥40	60	60	<345 ng/dL	low normal T
Hoyos 2012 [40]	i.m.	1,000 mg TU/6 weeks	4.5 months	49 ± 12 (SD)	33	34	388 ± 152 (SD) ng/dL	eugonadal, obese, sleep apnea
Kalichenko 2010 [41]	i.m.	1,000 mg TU/6 to 12 weeks	7.5 months	49 to 53	113	71	<345 ng/dL	low normal T, metabolic syndrome
Kenny 2004 [42]	i.m.	200 mg TE/3 weeks	3 months	81 ± 5 (SD)	6	5	410 ± 112 (SD) ng/dL	mild cognitive impairment
Sih 1997 [43]	i.m.	200 mg TC/2 weeks	12 months	65 ± 7 (SD)	17	15	233 ± 20 (SD) ng/dL	hypogonadal
Svartberg 2004 [44]	i.m.	250 mg TE/4 weeks	6 months	64 ± 6.5 (SD)	15	14	590 ± 164 (SD) ng/dL	eugonadal, COPD
Svartberg 2008 [45]	i.m.	1,000 mg TU/6 to 12 weeks	12 months	69 ± 5 (SD)	19	19	239 ± 54 (SD) ng/dL	hypogonadal
Sheffield-Moore 2011 [46]	i.m.	100 mg TE/week	5 months	73 ± 8 (SD)	8	8	<500 ng/dL	eugonadal
Tan 2013 [47]	i.m.	1,000 mg TU/8 weeks	12 months	53.8 ± 8.3 (SD)	56	58	<345 ng/dL	low normal T
*Basaria 2010 [7]	gel	100 to 150 mg T/day	6 months*	74 ± 5 (SD)	106	103	250 ± 57 (SD) ng/dL	hypogonadal, mobility limited
Brockenbrough 2006 [48]	gel	100 mg T/day	6 months	58.9 ± 14.9 (SD)	19	21	218 ± 64 (SD) ng/dL	hypogonadal, renal disease
Glintborg 2013 [49]	gel	50 to 100 mg T/day	6 months	62 to 72	20	18	<210 ng/dL	hypogonadal, obese
Hildreth 2013 [50]	gel	25 to 50 mg T/day	12 months	66.6 ± 5.8 (SD)	96	47	294 ± 38 (SD) ng/dL	hypogonadal
Jones 2011 [51]	gel	60 mg T/day	12 months	37 to 77	108	112	265 ± 75 (SD) ng/dL	hypogonadal, metabolic syndrome
Kaufman 2011 [52]	gel	20 to 80 mg T/day	6 months	53.6 ± 9.5 (SD)	234	40	mean =294 ng/dL	hypogonadal
Kenny 2010 [53]	gel	50 mg T/day	12 to 24 months	79.9 ± 7.3 (SD)	69	62	380 ± (SD) ng/dL	eugonadal, osteoporosis
Marin 1993 [54]	gel	125 mg T/day	9 months	56.7 ± 2.2 (SD)	11	10	434 ± 23 (SD) ng/dL	eugonadal, obese
Spitzer 2012 [55]	gel	100 to 300 mg T/day	3.5 months	55.1 ± 8.3 (SD)	70	70	248 ± 62 (SD) ng/dL	hypogonadal, erectile dysfunction
Srinivas-Shankar 2010 [56]	gel	50 mg T/day	6 months	73.7 ± 5.7 (SD)	138	136	313 ± 89 (SD) ng/dL	low normal T, frail
English 2000 [17]	patch	5 mg T/day	3 months	69 ± 2 (SD)	25	25	390 ± 22 (SD) ng/dL	eugonadal, stable angina
Malkin 2006 [57]	patch	5 mg T/day	12 months	63.1 ± 10.7 (SD)	37	39	400 ± 152 (SD) ng/dL	eugonadal, heart failure
Merza 2005 [58]	patch	5 mg T/day	6 months	63 ± 9 (SD)	20	19	242 ± 95 (SD) ng/dL	hypogonadal
Nair 2006 [59]	patch	5 mg T/day	24 months	61 to 72	27	31	bioavailable T <103 ng/dL	hypogonadal
Snyder 2001 [60]	patch	6 mg T/day	36 months	71.3 ± 5.8 (SD)	54	54	<475 ng/dL	eugonadal
Chapman 2009 [61]	oral	160 mg TU/day	12 months	78 ± 4 (SD)	11	12	541 ± 35 (SD) ng/dL	eugonadal, undernourished
Copenhagen study 1986 [62]^a^	oral	600 mg micronized T/day	8 to 62 months	24 to 79	134	87	not measured	alcoholic cirrhosis
Emmelot-Vonk 2008 [63]	oral	80 mg TU/day	6 months	67.1 ± 5.0 (SD)	120	117	316 ± 54 (SD) ng/dL	low normal T
Legros 2009 [64]	oral	80 to 240 mg TU/day	12 months	58.6 ± 5.7 (SD)	237	79	free T <7.5 ng/dL	hypogonadal

^a^Study was stopped early. COPD. chronic obstructive pulmonary disease; i.m., intramuscular; SD, standard deviation; T, testosterone; TC, testosterone cypionate; TE, testosterone enanthate; TU, testosterone undecanoate.

### Risk of CV events based on route of TRT

Of the 3,703 subjects, 2,114 receiving TRT had 131 CV-related events (6.2%), while 1,589 receiving placebo had 87 (5.5%) CV-related events. Two trials were stopped early, one because of AEs in the TRT arm [7] and one because a beneficial effect of TRT was ‘not foreseeable’ [32]. Additional file 3 shows a comprehensive list of the type and severity of the 218 CV events (if specified) in the TRT and placebo groups.

**Figure 2:**
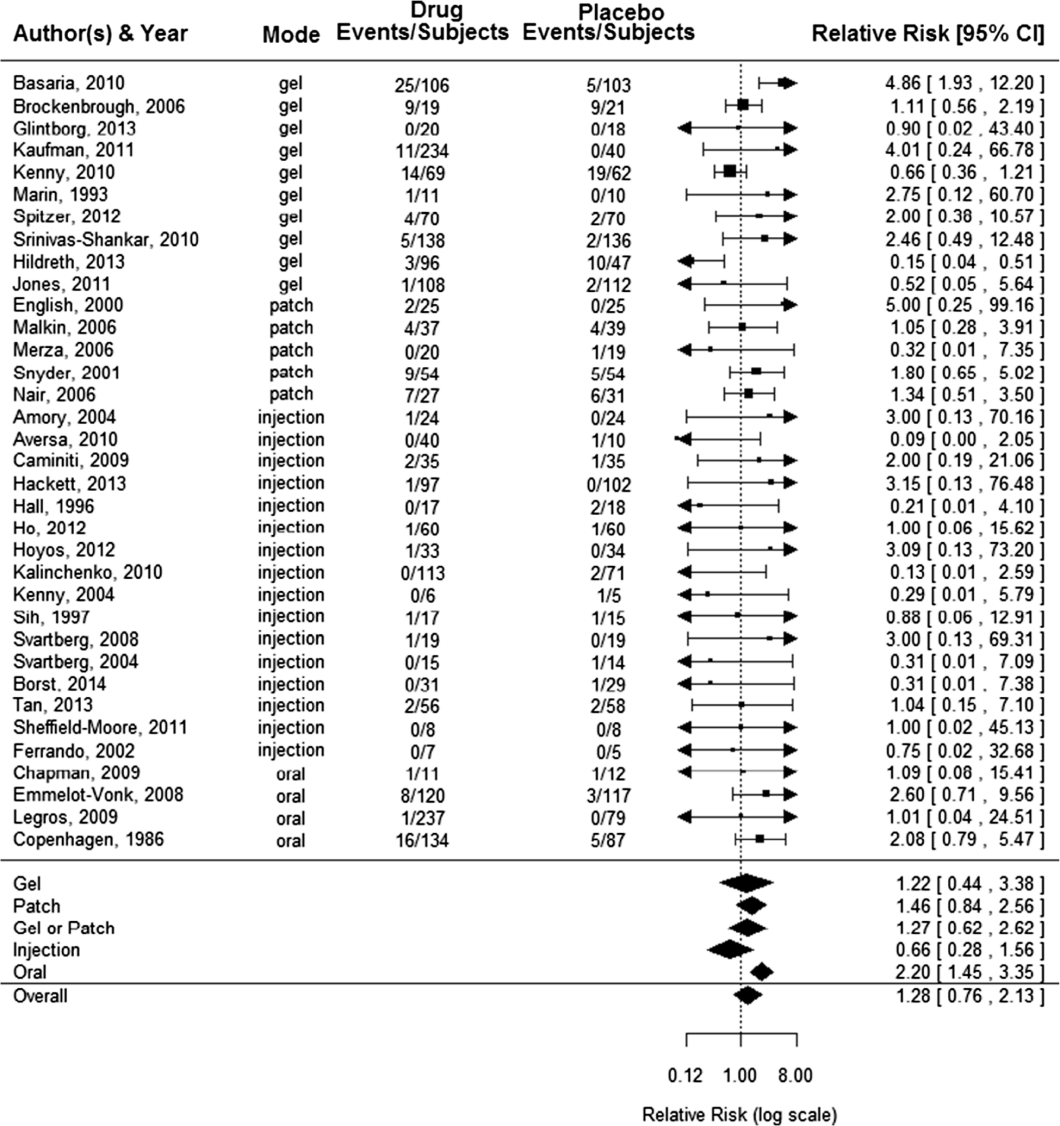
**Forest plot of all placebo-controlled randomized clinical trials (RCTs) reporting the pooled effect of testosterone replacement therapy (TRT) and the individual effects by TRT administration route on CV events.**

As shown in Figure 2, among patients receiving any form of TRT, the estimated RR for CV events was 1.28 (95% CI 0.76 to 2.13, *P* = 0.34) which was not statistically significant. However, CV event rates varied by mode of TRT administration. Specifically, oral TRT resulted in a significant increase in CV events (estimated RR = 2.20, 95% CI 1.45 to 3.35, *P* = 0.015). In contrast, neither intramuscular TRT (estimated RR = 0.66, 95% CI 0.28 to 1.56, *P* = 0.32) nor transdermal (patch or gel) TRT (estimated RR = 1.27, 95% CI 0.62 to 2.62, *P* = 0.48) significantly affected CV events.

Re-analysis of Xu *et al*. [10]: Using the same patient-weighted method [31], the estimated relative risk for CV AEs is 1.59 (95% CI = 0.90 to 2.57), *P* = 0.059, not quite statistically significant, whereas Xu *et al*. [10] using inverse variance weighted methods (against the advice of the *Cochrane Handbook*), reported a point estimate of 1.54 (95% CI =1.09 to 2.18).

### Route of TRT and elevation of serum T and DHT Levels

**Figure 3:**
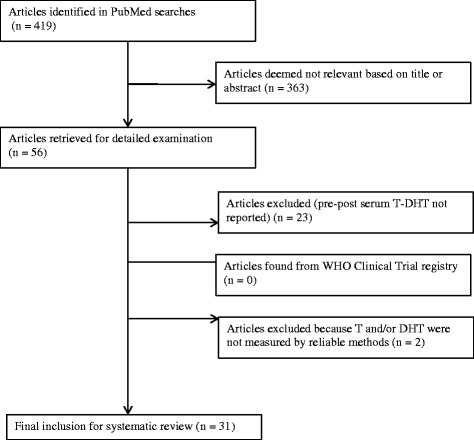
**Selection process for clinical trials reporting both serum testosterone (T) and dihydrotestosterone (DHT) concentrations before and after testosterone replacement therapy (TRT).**

As shown in Figure 3, the initial search yielded 419 publications, of which 56 were subjected to further scrutiny. We subsequently identified 31 unique publications that met our criteria and which included 1,176 men who received TRT (see Table 2). Elevation of serum DHT, but not T, was significantly affected by TRT administration mode (see Table 3). Specifically, intramuscular TRT elevated serum T and DHT to a roughly similar degree. In contrast, transdermal TRT elevated DHT to a significantly greater degree (5.46-fold, 95% CI 4.51 to 6.60) than intramuscular TRT (2.20-fold, (95% CI 1.74 to 2.77). Only four oral TRT studies were identified that reported both T and DHT, and the data were insufficient for statistical analysis. However, oral TRT appeared to produce a post-treatment serum T that was similar with other administration routes and very high post-treatment serum DHT values.

**Table 2:** **Characteristics of testosterone replacement therapy (TRT) trials reporting both serum testosterone (T) and dihydrotestosterone (DHT) concentrations before and after treatment**

**Author/Year**	**Study type**	**Mode**	**Dose**	**Duration**	**Age**	**Subjects in TRT group**	**Serum T at entry**	**Health status**
Amory 2004 [32]	RCT	i.m.	100 mg TE/week	36 months	71 ± 4 (SD)	24	302 ± 48 (SD) ng/dL	hypogonadal
Arver 1997 [65]	open-label	i.m.	266 mg TE/26 days	3 weeks	58 ± 10 (SD)	27	121 ± 100 (SD) ng/dL	hypogonadal
Bhasin 2012 [66]	RCT	i.m.	125 mg TE/week	5 months	40 ± 7 (SD)	12	519 ng/dL (mean)	eugonadal
Borst 2014 [34]	RCT	i.m.	125 mg TE/week	12 months	69.2 ± 8.0 (SD)	31	264 ± 92 (SD) ng/dL	hypogonadal
Lakshman 2010 [67]	RCT	i.m.	125 mg TE/week	5 months	65.6 ± 4.3 (SD)	11	581 ± 168 (SD) ng/dL	eugonadal
Raynaud 2008 [68]	open-label	i.m.	250 mg TE/3 weeks	12 months	41.8 ± 12.4 (SD)	32	43 ng/dL (mean)	hypogonadal
Shubert 2003 [69]	open-label	i.m.	250 mg TE/3 weeks	12 months	31.9 ± 2.5 (SD)	14	63.6 ng/dL ±14 (SD)	hypogonadal
Wang 2010 [70]	open-label	i.m.	750 mg TU/4 to 10 weeks	21 months	>18	117	320 ng/dL ±111 (SD)	low normal T
Brockenbrough 2006 [48]	RCT	gel	10 mg T/day	6 months	58.9 ± 14.9 (SD)	19	218 ± 64 (SD) ng/dL	hypogonadal, renal disease
Cherrier 2003 [71]	RCT	gel	50-100 mg T/day	6 months	34 to 70	12	320 ± 90 (SD) ng/dL	low normal T
Chiang 2007 [72]	RCT	gel	50 mg T/day	3 months	20 to 75	17	213 ± 158 (SD) ng/dL	hypogonadal
Dean 2004 [73]	open-label	gel	50 mg T/day	9 months	58.5 (mean)	257	247 ng/dL (mean)	hypogonadal
Di Luigi 2012 [74]	open-label	gel	50 mg T/day	1.25 month	31.3 ± 7.5 (SD)	10	72 ng/dL (mean)	hypogonadal
Juang 2014 [75]	RCT	gel	100 mg T/day	3.5 months	24 to 51	14	302 ± 37 (SD) ng/dL	hypogonadal, osteoporosis
Kenny 2010 [53]	RCT	gel	50 mg T/day	12 months	79.9 ± 7.3 (SD)	69	380 ± 179 (SD) ng/dL	eugonadal, osteoporosis
Marin 1993 [54]	RCT	gel	125 mg T/day	9 months	56.7 ± 2.2 (SD)	10	455 ± 23 (SD) ng/dL	eugonadal, obese
Mazer 2005 [76]	RCT	gel	59 mg/day	2 weeks	52.4 ± 12.2 (SD)	28	226 ± 110 (SD) ng/dL	hypogonadal
Page 2011 [77]	RCT	gel	75 mg T/day	6 months	>50	27	204 ng/dL (mean)	hypogonadal, BPH
Swerdloff 2000 [78]	open-label	gel	100 mg T/day	3 months	51.3 (mean)	76	280 ng/dL (mean)	hypogonadal
Wang 2000 [79]	no placebo group	gel	100 mg T/day	2 weeks	26 to 59	10	179 ± 41 (SD) ng/dL	hypogonadal
Wang 2011 [80]	open-label	gel	60 mg T/day	4 months	51.5 ± 12.7 (SD)	135	215 ± 84 (SD) ng/dL	hypogonadal
Ahmed 1988 [81]	no placebo group	patch	15 mg T/day	6 to 8 weeks	34 to 54	5	45 ± 12 (SD) ng/dL	hypogonadal
Bals-Pratch 1988 [82]	not stated	patch	10 to 15 mg T/day	14 months	31 to 37	7	189 ng/dL (mean)	hypogonadal
Behre 1999 [83]	open-label	patch	2.4 to 3.6 mg T/day	7 years	35.9 ± 9.8 (SD)	11	147 ± 37 (SD) ng/dL	hypogonadal
Cunningham 1989 [84]	placebo-controlled	patch	15 mg T/day	8 weeks	33 to 66	12	43 ± 11 (SD) ng/dL	hypogonadal
Mazer 2005 [76]	open-label	patch	5 mg T/day	2 weeks	28 to 71	28	215 ± 110 (SD) ng/dL	hypogonadal
Meikle 1992 [85]	not stated	patch	12.6 mg T/day	single dose	24 to 66	6	161 ± 27 (SD) ng/dL	hypogonadal
Raynaud 2008 [68]	open-label	patch	2.5 mg T/day	12 months	40.7 ± 10.5 (SD)	131	43 ng/dL (mean)	hypogonadal
Franchimont 1978 [86]		oral	120 to 240 mg TU/day	9 weeks	16 to 51	10	120 ng/dL (mean)	hypogonadal
Roth 2011 [87]	open-label	oral	400 mg TU/day	1 day	18 to52	11	405 ± 14 (SD) ng/dL	eugonadal
Schubert 2003 [69]	open-label	oral	160 mg TU/day	12 months	34.5 ± 3.9 (SD)	13	63.6 ng/dL ±14 (SD)	hypogonadal
Van Coevorden 1986 [88]	RCT	oral	240 mg TU/day	12 weeks	40 ± 11 (SD)	19	161 ± 86 (SD) ng/dL	hypogonadal, renal insufficiency

BPH, benign prostate hyperplasia; RCT, randomized clinical trial; SD, standard deviation; TE, testosterone enanthate; TU, testosterone undecanoate.

**Table 3:** **Change in serum testosterone (T) and dihydrotestosterone (DHT) when assessed by testosterone replacement therapy (TRT) administration route**

		**Testosterone**			**DHT**		
**Route of administration**	**Number of studies**	**Pre-treatment T (nmol/L) (95% CI)**	**Post-treatment T (nmol/L) (95% CI)**	**Pre-Post treatment fold increase in T (95% CI)**	**Pre-treatment DHT (nmol/L) (95% CI)**	**Post-treatment DHT (nmol/L) (95% CI)**	**Pre-Post treatment fold increase in DHT (95% CI)**
Intramuscular	8	9.27 (5.68 to 12.85)	23.11 (15.38 to 34.72)	2.91 (2.19 to 3.86)	1.02 (0.69 to 1.34)	1.62 (1.2 to 2.19)	2.20 (1.74 to 2.77)
Transdermal (patch and gel)	20	7.28 (6.09 to 8.42)	16.69 (12.62 to 21.98)	2.53 (1.83 to 3.50)	0.99 (0.78 to 1.20)	3.43 (2.37 to 4.98)	5.46 (4.51 to 6.60)
Gel	13	8.90 (7.67 to 10.13)	18.3 (15.18 to 23.12)	1.98 (1.70 to 2.30)	1.19 (0.93 to 1.46)	3.81 (2.57 to 5.63)	5.12 (4.07 to 6.45)
Patch	7	4.20 (2.78 to 5.23)	9.73 (4.01 to 23.62)	4.43 (2.99 to 6.54)	0.62 (0.36 to 0.88)	2.16 (0.68 to 6.87)	6.61 (3.08 to 14.16)
Oral^a^	4	6.66	21.88	2.80	0.90	3.92	4.46
		(14.05, 2.9, 5.6, 4.1)	(59.2, 5.70, 7.6, 14.96)	(4.20, 2.20, 1.4, 3.6)	(1.1, 1.8, 0.30, 0.41)	(9.89, 3.30, 1.13, 1.35)	9.0, 1.8, 3.8, 3.3

^a^Effects of oral TRT on T and DHT concentrations were not statistically analyzed because only four studies were identified that met our *a priori* inclusionary criteria, which resulted in sufficient data. For oral studies, the mean and individual values for each of the four studies are listed. Transdermal (patch or gel) TRT produces a greater elevation of serum DHT than intramuscular TRT. Means are adjusted for sample size.

## Discussion

This meta-analysis of 35 eligible studies and more than 3,700 patients receiving TRT is the largest consolidation of RCT data thus far. Our main finding is that no significant increase in CV event risk was noted among studies of various TRT administration routes when analyzed together. Further, when the risk of CV events was analyzed based on the mode of administration, only oral TRT was associated with elevated CV risk when compared with placebo. The increase in CV risk resulting from transdermal TRT and the decrease in CV risk seen with intramuscular TRT did not achieve statistical significance. A second important finding in this meta-analysis is that the oral and transdermal administration methods of TRT are associated with greater DHT elevations than intramuscular administration. Because there is emerging data demonstrating an association between elevated DHT (rather than serum T) and adverse CV events, these two findings may have important implications for our current understanding of the mechanisms of CV risk in TRT recipients.

### Mode of administration and CV risk

Our finding that there are varying CV risks based on the type of TRT formulation helps reconcile seemingly disparate observations across various studies regarding testosterone’s CV effects. While three prior meta-analyses suggested no significant increase in CV risk across TRT RCTs [4-6], a more recent meta-analysis by Xu *et al*. [10] indicated higher CV risk with TRT. The present meta-analysis is the most extensive thus far. Although we included all reported CV AEs in this meta-analysis, we have included newer studies exclusive to this review which may reflect less publication bias, more rigorous patient screening practices and more attention to the reporting of hard CV endpoints rather than nonspecific CV events that may have driven AE rates in previous studies.

The increased CV risk of the oral formulation subgroup is a novel finding in our analysis. *While no significant effects on CV risk were observed with either injected or transdermal TRT, the point estimates suggest that further research is needed to establish whether administration by these routes is protective or detrimental, respectively.* To the best of our knowledge, differing CV risk specific to varying testosterone formulations has not been previously reported.

### DHT elevation and increased CV risk

**Figure 4:**
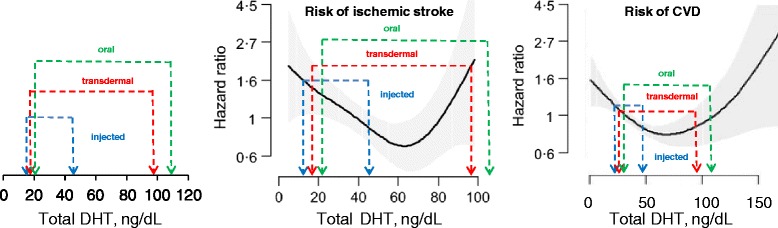
**Comparison of DHT levels after testosterone treatment with DHT levels that are associated with cardiovascular disease risk. Left panel**. Testosterone-induced elevation of DHT in the eight RCTs of testosterone injection, twenty RCTs of transdermal administration and four RCTs of oral testosterone administration shown in Table 2. Transdermal administration causes a greater elevation of serum DHT. **Center panel**. Data from panel 1 is overlayed on observational data from Shores *et al*. showing the relationship between serum DHT and 10 year risk of incident ischemic stroke in older men. The solid line represents the estimated hazard ratio (HR) and the shaded area depicts the 95% confidence interval. All models are adjusted for age (reprinted with permission from Shores *et al. Clin Endocrinol (Oxf)* 2014. doi: 10.1111/cen.12452 [22]. **Right panel**. Data from panel 1 is overlayed on observational data from Shores *et al*. showing the relationship between serum DHT and incident cardiovascular disease risk. The solid line represents the estimated hazard ratio (HR) and the shaded area depicts the 95% confidence intervals. All models are adjusted for age. (Reprinted with permission from Shores *et al. J Clin Endocrinol Metab* 2014, **99:**2061-2068. [23]). DHT, dihydrotestosterone; RCT, randomized controlled trial.

The greater elevation of DHT that occurs with oral or transdermal TRT may be due to the high expression of 5-α reductase in skin [20] and liver [21] in comparison to lower 5-α reductase in skeletal muscle [89]. The finding of differential DHT elevation may be critical to our understanding of adverse CV risk, because elevated serum DHT (not elevated T) has recently been found to be associated with CV risk in several observational studies. Shores *et al*. published two studies of 1,032 older men which reported significant associations between the serum DHT concentration and both the 10-year rate of incident ischemic stroke [23] and the 9-year rate of incident CV disease and all-cause mortality [16] (see Figure 4). Interestingly, similar relationships did not exist for serum total or free T, suggesting that CV risk resulting from TRT may result from the 5α-reduction of T to DHT. In both studies by Shores *et al*., the lowest risk was associated with a serum DHT concentration of approximately 60 ng/dL, while greater risk was associated with both higher and lower DHT concentrations.

In Figure 4, the left panel represents data from our meta-analysis showing the elevation of serum DHT with intramuscular, transdermal and oral TRT. In the center and right panels, we have superimposed that data on top of the previously published data from the two papers by Shores *et al*. Taken together, these data appear to indicate that intramuscular TRT elevates the serum DHT concentration into a range that is associated with *reduced* CV disease (CVD) and stroke risks. In contrast, transdermal and oral TRT appear to elevate serum DHT into a range that is associated with unchanged CVD risk and *increased* ischemic stroke risk.

### Limitations

Reporting of AEs may be open to interpretation and so may vary somewhat among trials. Using the most serious CV events (stroke, myocardial infarction, and CV-related death) might be more unambiguous. Because of very long follow-up periods, such events are common enough to assess in observational studies [16,22,23]. However, due to shorter study duration, serious CV events are not common enough to study in clinical trials of TRT. As a result, our analyses are based on all CV events, serious or not.

The data on oral TRT must be interpreted with caution, since only four studies met the inclusion criteria. Of those, two had very low rates of CV events in both the treated and placebo groups [61,64] and one study had very high post-treatment serum T concentrations [32], possibly due to the presence of liver disease in the study subjects. The latter study was not included in the analysis of TRT-induced elevations of T/DHT because DHT was not measured. However, among the four studies analyzed for T/DHT, there was considerable variation in serum concentrations. Variation may result from the fact that serum T concentrations are not sustained following oral TRT and the time of blood acquisition is therefore critical.

Two studies included in the analysis of CV risk were stopped early. One study of oral TRT was stopped because of lack of evidence for efficacy unrelated to CV [32] and one study of gel TRT was stopped early for excess CV events in the group receiving testosterone [7]. The first study, whose stopping was uninfluenced by CV has no bias associated with early stopping. The second, may actually be associated with a slight bias estimate away from the null, actually strengthening the null conclusion. There is no way to adjust for this without serial patient level data and the exact stopping rules used.

Interpretation of the data on TRT-induced elevations of T and DHT may be limited by the fact that DHT was assayed by several methods in the included studies. The latter include mass spectroscopy (MS) based methods and various radioimmunoassays (RIAs). MS-based assays provide highly accurate measurements of DHT. RIAs are specific for DHT [90] but the values are somewhat higher than those obtained with MS-based assays [91]. The enzyme-linked immunosorbant assay (EIA) for DHT is not valid as we have recently shown [24] and studies using this method were excluded. The current analysis is based on clinical trials that have a high rate of compliance. An additional limitation in extending our findings to a clinical setting is that compliance may be lower. Schoenfeld *et al*. have shown that TRT gel adherence is only 37.4% at six months [92]. Similarly, Donatucci *et al*. [93] reported that at three months, adherence to transdermal TRT was 52% and adherence to injected TRT was 32%.

#### Potential cardiovascular benefits of testosterone

Although this paper encompasses a discussion of adverse CV risk of TRT, assessment of the CV risk–to-benefit should be considered. Numerous studies have demonstrated positive CV effects of TRT. English *et al*. [17] have shown that, in men with stable angina, treatment with low-dose T (5 mg/day by patch) for 12 weeks caused a significant 17% increase in time to 1-mm ST segment depression during treadmill exercise testing. Stout *et al*. [94] have shown that TRT administration to men with chronic heart failure increases maximal oxygen consumption (VO_2max_) and improves physical performance. Toma *et al*. [18] published a meta-analysis of the four studies showing that TRT improved exercise capacity in heart failure patients. Empen *et al*. [95] reported that T deficiency is associated with impaired arterial flow-mediated dilation (FMD), a marker of vascular endothelial function. Cardiovascular improvement with TRT is thought to result from increased coronary blood flow, peripheral vasodilation, positive remodeling of skeletal muscle and reduced insulin resistance, without marked effects on left ventricular ejection fraction [18].

## Conclusions

The potential CV risks of TRT are currently being debated. This updated meta-analysis indicates oral TRT produces increased CV risk, while TRT administered by all routes may cause an increase in CV adverse events, but the effect is not statistically significant. On the latter point, a definitive answer awaits further clinical trials. More studies are also needed to assess whether increased CV risk occurs with the transdermal formulations and decreased CV risk with the intramuscular formulation. This early indicator that intramuscular T may be safer than transdermal TRT may be surprising, considering that intramuscular TRT doses are typically several-fold higher than transdermal doses. However, our data indicate that transdermal TRT produces a significantly greater elevation of serum DHT than intramuscular T, possibly due to the expression of 5-alpha reductase in the skin. Interestingly, serum DHT concentrations following intramuscular TRT correspond to DHT levels that are associated with reduced CV risk in other large observational studies, suggesting that: 1) CV risks of TRT administration may result from excessive elevation of serum DHT; and 2) intramuscular TRT may produce less CV risk than transdermal or oral TRT. Given our unique findings, future RCTs, meta-analyses and retrospective database studies evaluating the health risks associated with TRT should carefully control for the change in serum DHT and evaluate the TRT administration route as potential confounding factors in their data analysis.

## Additional files

Additional file 1:
**PRISMA 2009 Checklist.**
Additional file 2:
**ICD 10 codes for cardiovascular disease.**
Additional file 3:
**Listing of CV events in RCTs selected for analysis of CV events.**
Additional file 4:
**Quality assessment for RCTs reporting the effect of TRT on CV events.**
Additional file 5:
**Quality assessment for trials reporting elevation of serum T and DHT following TRT.**
Additional file 6:
**Inclusion of RCTs for analysis of CV events following attempts to obtain additional information from authors.**
