# Supplementary material for: Cardiovascular risks and elevation of serum DHT vary by route of testosterone administration: a systematic review and meta-analysis
Source: BMC Med. 2014 Nov 27;12:211. doi: 10.1186/s12916-014-0211-5 (PMC4245724; doi:10.1186/s12916-014-0211-5)
Supplement: Additional file 2: — ICD 10 codes for cardiovascular disease. [file 12916_2014_211_MOESM2_ESM.docx]

**Online File 2.** ICD 10 Codes for cardiovascular disease

[1 I00–I99 – Diseases of the circulatory system](http://en.wikipedia.org/wiki/ICD-10_Chapter_IX:_Diseases_of_the_circulatory_system#I00.E2.80.93I99_.E2.80.93_Diseases_of_the_circulatory_system)

[1.1 (I00–I02) Acute rheumatic fever](http://en.wikipedia.org/wiki/ICD-10_Chapter_IX:_Diseases_of_the_circulatory_system#.28I00.E2.80.93I02.29_Acute_rheumatic_fever)

[1.2 (I05–I09) Chronic rheumatic heart diseases](http://en.wikipedia.org/wiki/ICD-10_Chapter_IX:_Diseases_of_the_circulatory_system#.28I05.E2.80.93I09.29_Chronic_rheumatic_heart_diseases)

[1.3 (I10–I15) Hypertensive diseases](http://en.wikipedia.org/wiki/ICD-10_Chapter_IX:_Diseases_of_the_circulatory_system#.28I10.E2.80.93I15.29_Hypertensive_diseases)

[1.4 (I20–I25) Ischemic heart diseases](http://en.wikipedia.org/wiki/ICD-10_Chapter_IX:_Diseases_of_the_circulatory_system#.28I20.E2.80.93I25.29_Ischemic_heart_diseases)

[1.5 (I26–I28) Pulmonary heart disease and diseases of pulmonary circulation](http://en.wikipedia.org/wiki/ICD-10_Chapter_IX:_Diseases_of_the_circulatory_system#.28I26.E2.80.93I28.29_Pulmonary_heart_disease_and_diseases_of_pulmonary_circulation)

[1.6 (I30–I52) Other forms of heart disease](http://en.wikipedia.org/wiki/ICD-10_Chapter_IX:_Diseases_of_the_circulatory_system#.28I30.E2.80.93I52.29_Other_forms_of_heart_disease)

- - - [1.6.1 Pericardium](http://en.wikipedia.org/wiki/ICD-10_Chapter_IX:_Diseases_of_the_circulatory_system#Pericardium)
    - [1.6.2 Endocardium (including heart valves)](http://en.wikipedia.org/wiki/ICD-10_Chapter_IX:_Diseases_of_the_circulatory_system#Endocardium_.28including_heart_valves.29)
    - [1.6.3 Myocardium / cardiomyopathy](http://en.wikipedia.org/wiki/ICD-10_Chapter_IX:_Diseases_of_the_circulatory_system#Myocardium_.2F_cardiomyopathy)
    - [1.6.4 Other](http://en.wikipedia.org/wiki/ICD-10_Chapter_IX:_Diseases_of_the_circulatory_system#Other)

[1.7 (I60–I69) Cerebrovascular diseases](http://en.wikipedia.org/wiki/ICD-10_Chapter_IX:_Diseases_of_the_circulatory_system#.28I60.E2.80.93I69.29_Cerebrovascular_diseases)

[1.8 (I70–I79) Diseases of arteries, arterioles and capillaries](http://en.wikipedia.org/wiki/ICD-10_Chapter_IX:_Diseases_of_the_circulatory_system#.28I70.E2.80.93I79.29_Diseases_of_arteries.2C_arterioles_and_capillaries)

[1.9 (I80–I89) Diseases of veins, lymphatic vessels and lymph nodes, not elsewhere classified](http://en.wikipedia.org/wiki/ICD-10_Chapter_IX:_Diseases_of_the_circulatory_system#.28I80.E2.80.93I89.29_Diseases_of_veins.2C_lymphatic_vessels_and_lymph_nodes.2C_not_elsewhere_classified)

[1.10 (I95–I99) Other and unspecified disorders of the circulatory system](http://en.wikipedia.org/wiki/ICD-10_Chapter_IX:_Diseases_of_the_circulatory_system#.28I95.E2.80.93I99.29_Other_and_unspecified_disorders_of_the_circulatory_system)
