# Supplementary material for: Cardiovascular risks and elevation of serum DHT vary by route of testosterone administration: a systematic review and meta-analysis
Source: BMC Med. 2014 Nov 27;12:211. doi: 10.1186/s12916-014-0211-5 (PMC4245724; doi:10.1186/s12916-014-0211-5)
Supplement: Additional file 3: — Listing of CV events in RCTs selected for analysis of CV events. [file 12916_2014_211_MOESM3_ESM.doc]

**Online file 3.** Listing of CV events in RCTs selected for analysis of CV events. T = testosterone treated group, P = placebo

|  | **event** | **T** | **P** | **comment** |
| --- | --- | --- | --- | --- |
| Amory 2004 | cerebral hemorrhage | 1 | 0 |  |
| Aversa 2010 | acute myocardial infarction | 0 | 1 |  |
| Borst 2014 | syncope due to bradycardia | 0 | 1 |  |
| Caminiti 2009 | worsening of heart failure – hospitalization  worsening of heart failure – no hospitalization | 1  1 | 1  0 |  |
| Ferrando 2002 | none | 0 | 0 | Authors confirmed to Borst et al that no CV events occurred |
| Hackett 2013 | Worsening of atrial fibrillation | 1 | 0 | Authors supplied events table to Borst et al. |
| Hall 1996 | hypertension  cerebrovascular accident | 0  0 | 1  1 |  |
| Ho 2011 | died of myocardial infarction | 1 | 1 |  |
| Hoyos 2012 | unspecified CV event | 1 | 0 |  |
| Kalinchenko 2010 | angina  died of myocardial infarction | 0  0 | 1  1 |  |
| Kenny 2004 | cerebrovascular accident | 0 | 1 |  |
| Sih 1997 | atrial fibrillation with congestive heart failure  stroke | 0  0 | 1  1 |  |
| Svartberg 2004 | death due to probable myocardial infarction | 0 | 1 |  |
| Svartberg 2008 | death due to cardiac arrhythmia | 1 | 0 |  |
| Sheffield-Moore 2011 | none | 0 | 0 | Authors confirmed to Borst et al that no CV events occurred |
| Tan 2013 | Myocardial infarction | 2 | 2 |  |
| Basaria 2010 | acute coronary syndrome and chest pain  syncope  myocardial infarction  angioplasty and coronary artery bypass  peripheral edema  ectopy on ECG  left ventricular strain pattern during exercise test  ST segment depression during exercise testing  hypertension and atrial fibrillation  hypertension  chest pain  atrial fibrillation  stroke  tachycardia with fatigue  tachycardia  death of suspected myocardial infarction  exacerbation of heart failure  arrhythmia and ectopy on ECG before exercise test  carotid bruit and plaque identified by ultrasound | 2  2  2  1  5  1  1  1  1  2  1  2  1  1  0  1  1  0  0 | 0  1  0  0  0  0  0  0  0  1  0  0  0  0  1  0  0  1  1 | Several subjects had CV events of procedures that were attributed to prior conditions and were not counted. |
| Brockenbrough 2006 | CV events involving death  CV events not involving death  access thrombosis | 3  4  2 | 1  2  6 | CV death in the P group was from stroke |
| Glintborg 2013 | none | 0 | 0 | Authors confirmed to Borst et al that no CV events occurred |
| Hildreth 2013 | Acute coronary syndrome  Arrhythmia  Aortic aneurysm  Syncope/presyncope | 1  0  0  2 | 3  4  1  2 |  |
| Jones 2011 | died of myocardial infarction  unspecified CV events | 0  5 | 1  11 | Unspecified CV events are not given as a number but may be inferred from percentages reported. |
| Kaufman 2011 | died of myocardial infarction  unspecified CV events | 1  10 | 0  0 |  |
| Kenny 2010 | cerebrovascular accident  shortness of breath  death due to CAD and alcohol  chest pain  acute myocardial infarction  coronary bypass surgery  diaphoresis and disorientation  death – unexplained  MI with coronary bypass surgery  death due to cerebrovascular accident  heart failure  exacerbation of heart failure  syncope  angioplasty  death due to arrhythmia  transient ischemic attack  ECG suggest leaky heart valve  ascending aortic aneurysm  death due to transient ischemic attack and infection  admission for chest tightness | 1  1  1  0  0  2  0  0  0  1  2  1  2  1  0  1  1  0  0  0 | 3  1  0  2  2  1  1  1  1  0  2  0  0  1  1  0  0  1  1  1 | CV adverse event table supplied by authors to Borst et al. |
| Marin 1993 | splanchnic venous thrombosis | 1 | 0 | Identification of treatment arm supplied by authors to Xu et al. |
| Spitzer 2012 | CV events requiring hospitalization  CV events not requiring hospitalization | 1  3 | 0  0 |  |
| Srinivas-Shankar | acute myocardial infarction  death due to ruptured aneurysm  angina  pulmonary embolism  died of constrictive pericarditis  abdominal aneurism with surgery  heart failure | 0  0  1  1  1  1  1 | 1  1  0  0  0  0  0 |  |
| English 2000 | myocardial infarction (MI)  early elective coronary angioplasty  MI while awaiting coronary revascularization | 1  1  1 | 0  0  0 | 1^st^ 2 events are listed as withdrawals; the 3^rd^ event is listed under “Safety” and is assumed to be one of the 1^st^ two events. |
| Malkin 2006 | arrhythmia  stroke  unstable angina  hospitalized | 0  1  1  2 | 2  0  0  2 |  |
| Merza 2005 | angina | 0 | 1 |  |
| Nair 2006 | ascending aorta dilation  CAD stent placement  chest pain  coronary artery bypass graft  coronary artery disease  ventricular ectopic beats  hypotension  phlebitis  triple bypass surgery | 1  0  0  1  2  1  0  1  1 | 2  1  1  0  0  0  1  1  0 |  |
| Snyder 2001 | myocardial infarction  coronary bypass graft surgery  arrhythmia  vascular events | 2  2  3  2 | 1  2  1  1 |  |
| Chapman 2009 | death from myocardial infarction  hospitalization for myocardial infarction | 1  0 | 0  1 | 1 chest pain in P group not counted |
| Copenhagen study 1986 | death from bleeding esophageal varicies  death from acute myocardial infarction  thrombosis | 12  1  3 | 5  0  0 |  |
| Emmelot-Vonk 2008 | CV complaints | 8 | 3 |  |
| Legros 2009 | death from arrhythmia and cardiac enlargement | 1 | 0 |  |
