# Supplementary material for: Cardiovascular risks and elevation of serum DHT vary by route of testosterone administration: a systematic review and meta-analysis
Source: BMC Med. 2014 Nov 27;12:211. doi: 10.1186/s12916-014-0211-5 (PMC4245724; doi:10.1186/s12916-014-0211-5)
Supplement: Additional file 4: — Quality assessment for RCTs reporting the effect of TRT on CV events. [file 12916_2014_211_MOESM4_ESM.docx]

**Online File 4**. Quality assessment for RCTs reporting the effect of TRT on CV events

|  | **randomized** | **Placebo-controlled** | **Subjects blinded** | **Investigators blinded** | **CV AE assessor blinded** | **Criteria for CV events pre-specified** | **Table of CV events by study arm** |
| --- | --- | --- | --- | --- | --- | --- | --- |
| Amory 2004 | yes | yes | yes | yes | not reported | NR | no |
| Aversa 2010 | yes | yes | yes | yes | not reported | NR | no |
| Borst 2014 | yes | yes | yes | yes | yes | no | no |
| Caminiti 2009 | yes | yes | yes | yes | not reported | NR | no |
| Ferrando 2002 | yes | yes | yes | yes | not reported | NR | no |
| Hackett 2013 | yes | yes | yes | yes | not reported | NR | no |
| Hall 1996 | yes | yes | yes | yes | not reported | NR | no |
| Ho 2011 | yes | yes | yes | yes | yes | NR | no |
| Hoyos 2012 | yes | yes | yes | yes | not reported | NR | yes |
| Kalinchenko 2010 | yes | yes | yes | yes | not reported | NR | no |
| Kenny 2004 | yes | yes | yes | yes | not reported | NR | no |
| Sih 1997 | yes | yes | yes | yes | not reported | NR | no |
| Svartberg 2004 | yes | yes | yes | yes | not reported | NR | no |
| Svartberg 2008 | yes | yes | yes | yes | not reported | NR | no |
| Sheffield-Moore 2011 | yes | yes | yes | yes | not reported | NR | no |
| Tan 2013 | yes | yes | yes | yes | not reported | NR | yes |
| Basaria 2010 | yes | yes | yes | yes | yes | yes | Yes |
| Brockenridge 2006 | yes | yes | yes | yes | not reported | NR | Yes |
| Glintborg 2013 | yes | yes | yes | yes | not reported | NR | no |
| Hildreth 2013 | yes | yes | yes | yes | not reported | NR | yes |
| Jones 2011 | yes | yes | yes | yes | yes | NR | no |
| Kaufman 2011 | yes | yes | yes | yes | not reported | NR | yes |
| Kenny 2010 | yes | yes | yes | yes | not reported | NR | no |
| Marin 1993 | yes | yes | yes | yes | not reported | NR | no |
| Spritzer 2012 | yes | yes | yes | yes | not reported | NR | yes |
| Srinivas-Shankar 2010 | yes | yes | yes | yes | not reported | NR | no |
| English 2000 | yes | yes | yes | yes | not reported | NR | no |
| Malkin 2006 | yes | yes | yes | yes | not reported | NR | yes |
| Merza 2005 | yes | yes | yes | yes | not reported | NR | no |
| Snyder 2001 | yes | yes | yes | yes | not reported | NR | no |
| Chapman 2009 | yes | yes | yes | yes | not reported | NR | no |
| Copenhagen study 1986 | yes | yes | yes | yes | yes | yes | yes |
| Emmelot-Vonk 2008 | yes | yes | yes | yes | yes | yes | yes |
| Legros 20009 | yes | yes | yes | yes | not reported | NR | no |
