# Supplementary material for: Cardiovascular risks and elevation of serum DHT vary by route of testosterone administration: a systematic review and meta-analysis
Source: BMC Med. 2014 Nov 27;12:211. doi: 10.1186/s12916-014-0211-5 (PMC4245724; doi:10.1186/s12916-014-0211-5)
Supplement: Additional file 5: — Quality assessment for trials reporting elevation of serum T and DHT following TRT. [file 12916_2014_211_MOESM5_ESM.docx]

**Online file 5**. Quality assessment for trials reporting elevation of serum T and DHT following TRT. LC-MS/MS = liquid chromatography tandem mass spectroscopy, GC-MS = gas chromatography mass spectroscopy, Cobas = Cobas electrochemoluminescence immunoassay, RIA = radio immunoassay, HPLC = high performance liquid chromatography

|  | **Type of study** | **mode of T administration** | **Method for T measurement** | **Method for DHT measurement** | **Are baseline T and DHT concentrations in the expected range?** | **T and DHT measured before and after T-treatment?** | **Were some values read from a graph?** |
| --- | --- | --- | --- | --- | --- | --- | --- |
| Amory 2004 | RCT | injected | not reported | RIA | yes | yes | no |
| Arver 1997 | open-label | injected | not reported | not reported | yes | yes | no |
| Bhasin 2012 | RCT | injected | LC-MS/MS | LC-MS/MS | yes | yes | yes |
| Borst 2014 | RCT | injected | Cobas | GC-MS | yes | yes | no |
| Lakshman 2010 | RCT | injected | LC-MS-MS | LC-MS/MS | yes | yes | yes |
| Raynaud 2008 | open-label | injected | time-resolved fluoro- immunoassay | RIA | yes | yes | yes |
| Schubert 2003 | open-label | injected | RIA | RIA | yes | yes | no |
| Wang 2000 | open-label | injected | LC-MS/MS | LC-MS/MS | yes | yes | no |
| Brockenbrough 2006 | RCT | gel | fluoro-immuno assay | fluoro-immuno assay | yes | yes | no |
| Cherrier 2003 | RCT | gel | RIA | RIA | yes | yes |  |
| Chiang 2003 | RCT | gel | not reported | not reported | yes | yes | yes |
| Dean 2004 | open-label | gel | RIA | RIA | yes | yes | no |
| Di Luigi 2012 | open-label | gel | immunoradiometric assay | RIA | yes | yes | no |
| Juang 2014 | RCT | gel | Cobas | LC-MS | yes | yes | no |
| Kenny 2010 | RCT | gel | not reported | not reported | yes | yes | no |

| Marin 1993 | RCT | gel | immunoradiometric assay | not reported | yes | yes | no |
| --- | --- | --- | --- | --- | --- | --- | --- |
| Mazer 2005 | not reported | gel | immunoradiometric assay | immunoradiometric assay | yes | yes | no |
| Page 2011 | RCT | gel | LC-MS | LC-MS | yes | yes | yes |
| Swerdloff 2000 | open-label | gel | RIA | RIA | yes | yes | yes |
| Wang 2000 | no placebo group | gel | RIA | RIA | yes | yes | yes |
| Wang 2011 | open-label | gel | LC-MS/MS | LC-MS/MS | yes | yes | yes |
| Ahmed 1998 | no placebo group | patch | RIA | RIA | yes | yes |  |
| Bals-Pratch | not stated | patch | RIA | RIA following cellite chromatography | yes | yes | no |
| Behre 1999 | open-label | patch | RIA following HPLC | RIA following HPLC | yes | yes | yes |
| Cunningham 1989 | placebo controlled | patch | RIA following paper chromatography | RIA following paper chromatography | yes | yes | no |
| Mazer 2005 | not reported | patch | immunoradiometric assay | immunoradiometric assay | yes | yes | no |
| Meikle 1992 | not reported | patch | not reported | RIA following solvent extraction | yes | yes | no |
| Raynaud 2008 | open-label | patch | time-resolved fluoro- immunoassay | RIA | yes | yes | yes |
| Franchimont 1978 | Placebo controlled, blinding status not reported | oral | RIA | RIA | yes | yes | no |
| Roth 2010 | open-label | oral | LC-MS/MS | LC-MS/MS | yes | yes | no |
| Schubert 2003 | open-label | oral | RIA | RIA | yes | yes | no |
| Van Coevorden 1986 | RCT | oral | RIA | RIA | yes | yes | no |
