# Supplementary material for: Cardiovascular risks and elevation of serum DHT vary by route of testosterone administration: a systematic review and meta-analysis
Source: BMC Med. 2014 Nov 27;12:211. doi: 10.1186/s12916-014-0211-5 (PMC4245724; doi:10.1186/s12916-014-0211-5)
Supplement: Additional file 6: — Inclusion of RCTs for analysis of CV events following attempts to obtain additional information from authors. [file 12916_2014_211_MOESM6_ESM.docx]

**Online file 6**. Inclusion of RCTs for analysis of CV events following attempts to obtain additional information from authors

|  | action |
| --- | --- |
| Chapman 2009 | AEs are listed, but not by study arm. The study was included following written confirmation from the authors of 1 death from MI in the T-treated group and 1 in the placebo-treated group |
| Ferrando 2002 | Study was included following written confirmation from the authors that no CV events occurred during the study |
| Glintborg 2013 | Study was included following written confirmation from the authors that no CV events occurred during the study |
| Hackett 2013 | Study was included after a table of adverse events was sent by the authors |
| Kenny 2010 | Study was included after a table of adverse events was sent by the authors |
| Legros 2009 | Study listed only deaths by study arm. Other adverse events were not listed by arm. Study was included using 1 death by MI in the T-treated group after authors did not provide additional information. |
| Sheffield-Moore | Study was included following written confirmation from the authors that no CV events occurred during the study |
